# Calcineurin Activation by Prion Protein Induces Neurotoxicity via Mitochondrial Reactive Oxygen Species

**DOI:** 10.1155/2021/5572129

**Published:** 2021-08-06

**Authors:** Ji-Hong Moon, Jeong-Min Hong, Sang-Youel Park

**Affiliations:** Biosafety Research Institute, College of Veterinary Medicine, Jeonbuk National University, Iksan, Jeonbuk 54596, Republic of Korea

## Abstract

Prion diseases are caused by PrPsc accumulation in the brain, which triggers dysfunctional mitochondrial injury and reactive oxygen species (ROS) generation in neurons. Recent studies on prion diseases suggest that endoplasmic reticulum (ER) stress induced by misfolding proteins such as misfolded prion protein results in activation of calcineurin. Calcineurin is a calcium-related protein phosphatase of type 2B that exists in copious quantities in the brain and acts as a critical nodal component in the control of cellular functions. To investigate the relationship between calcineurin and intracellular ROS, we assessed the alteration of CaN and ROS induced by prion peptide (PrP) 106-126. Human prion peptide increased mitochondrial ROS by activating calcineurin, and the inhibition of calcineurin activity protected mitochondrial function and neuronal apoptosis in neuronal cells. These results suggest that calcineurin plays a pivotal role in neuronal apoptosis by mediating mitochondrial injury and ROS in prion diseases.

## 1. Introduction

Prion diseases are fatal neurodegenerative conditions that cause protein accumulation in the brain. This accumulation is a key pathogenic mechanism of various neurodegenerative diseases, including Parkinson's disease and Alzheimer's disease [1, 2]. In these diseases, the misfolded prion protein (PrPsc) is incorporated into the fibrillary beta-sheet-rich structures, known as amyloid plaques, that have been associated with numerous protein misfolding disorders [3]. Although there is literature that confirms that PrPsc strain induces neuronal apoptosis in the brain [4–6], the molecular mechanisms and signaling cascades that result in neuronal apoptosis remain unclear [7–9].

The synthetic human prion peptide (PrP) 106-126 originates in PrP molecules found in numerous species, preserves features of the physiological and pathogenic abilities of PrPsc, and can trigger neuronal apoptosis [10–12]. Amino acids 106-126 of the PrP sequence could replicate biological features of PrPsc *in vitro*, such as amyloidogenesis, and its neurotoxic as well as its gliotrophic effects [13–15]. Recent studies have used neurotoxic PrP peptides such as PrP 106-126 to test the neuroprotective effect of autophagy-inducing agents [16–18].

Accumulated evidence suggests that cellular damage, such as oxidative stress caused by free radicals and various proteins, is dynamically implicated in the cellular apoptosis associated with several neurodegenerative diseases [19–23]. There is also evidence that prion-mediated neuronal cell death is related to oxidative stress [24–26]. Mitochondria play an essential role in regulating apoptosis and the production of reactive oxygen species in many neurodegenerative disorders, including prion diseases [27–29]. In *in vitro* models, endoplasmic reticulum (ER) stress resulting from the accumulation of misfolded prion proteins is associated with mitochondrial dysfunction and ROS production [30–32]. Over the past decade, numerous studies have used mitochondrion-targeted probe MitoSOX for mitochondrial ROS detection [33, 34]. Dichlorodihydrofluorescein diacetate (DCFH-DA) is typically used for detecting intracellular ROS [35]. To determine the role of cellular ROS and mitochondrial ROS in prion-mediated neuronal apoptosis, we performed experiments to detect cellular ROS and mitochondrial ROS.

Impaired calcium signaling caused by the accumulation of a misfolded prion protein triggers crosstalk between the endoplasmic reticulum and the mitochondria as a means of counteracting stress [36–38]. Our previous studies suggested that PrP 106-126 increases intracellular calcium levels and calcineurin (CaN) activity in neurons [39, 40]. Other researchers have shown that increased calcium levels are associated with the mitochondrial apoptotic pathway in neurodegenerative diseases that involve prions [31, 41]. When mitochondrial depolarization occurs with a sustained cytosolic rise in Ca^2+^, the cytosolic phosphatase activity of the serine/threonine phosphatase CaN is activated [42]. Activated CaN dephosphorylates cytoplasmic NFAT, which exposes its nuclear localization sequence and leads to rapid nuclear import [43]. CaN is a calcium-mediated type 2B protein phosphatase and a crucial nodal factor in controlling cellular functions [44]. CaN is highly abundant in the brain and has been implicated in the regulation of synaptic plasticity, memory, and neuronal death [45]. The relationship between prion-mediated ROS and CaN activation has, until now, not been described. In this study, we investigated what kind of ROS is generated by a prion peptide and the relationship between CaN and intracellular ROS in prion *in vitro* models.

## 2. Materials and Methods

### 2.1. Cell Culture

The human neuroblastoma cell line SK-N-SH was obtained from the American Type Culture Collection (ATCC, Rockville, MD, USA). The cell culturing method has been described previously in detail [39]. The cells were cultured in Minimum Essential Medium (HyClone Laboratories, Logan, UT, USA) containing 10% FBS (Gibco, Grand Island, NY, USA) and gentamycin (0.1 mg/mL) in a humidified incubator at 37°C with 5% CO_2_.

### 2.2. Chemical and PrP (106-126) Treatment

Synthetic prion peptides PrP (106-126) (sequence, Lys-Thr-Asn-Met-Lys-His-Met-Ala-Gly-Ala-Ala-Ala-Ala-Gly-Ala-Val-Val-Gly-Gly-Leu-Gly) and scrambled PrP (106-126) (sequence, Asn-Gly-Ala-Lys-Ala-Leu-Met-Gly-Gly-His-Gly-Ala-Thr-Lys-Val-Met-Val-Gly-Ala-Ala-Ala) were synthesized by Peptron (Seoul, Korea) [20]. The PrP peptides were dissolved in sterile dimethyl sulfoxide (DMSO) at a concentration of 10 mM (stock) and stored at -20°C.

The stock solution of FK506 (10 mM; F4679, Sigma-Aldrich, St. Louis, MO, USA) was dissolved in DMSO. The stock solution of *N*-acetyl-L-cysteine (NAC, 1 M; A7250, Sigma-Aldrich), diethyldithiocarbamate (DDC, 100 mM; Sigma-Aldrich), and 3-amino-1,2,4-triazole (AT, 500 mM; Sigma-Aldrich) was dissolved in distilled water.

### 2.3. Thioflavin-T Binding Assay

Cells in the logarithmic phase were collected and cultured in 6-well plates at 3 × 10^5^ cells/well. Binding of ThT to PrP 106-126 and amyloid *β* fibrils was assayed by adding 20 *μ*M ThT (T3516, Sigma-Aldrich, St. Louis, MO, USA) to a solution of fibrils. Fluorescence was monitored using a SpectraMax M2 (Molecular Devices) with excitation and emission slit widths set to 5 nm, respectively. Spectra were obtained by scanning the fluorescence emission from 430 to 500 nm, with excitation at 442 nm. Fluorescence images were obtained using fluorescence microscopy (Nikon Eclipse 80i). An image was evaluated using the NIS-Elements F ver4.60 Imaging software.

### 2.4. Annexin V Assay

Cells in the logarithmic phase were collected and cultured in a 24-well plate at 4 × 10^4^ cells/well. Cell survival was evaluated using an annexin V assay kit (Santa Cruz Biotechnology, CA, USA) following the manufacturer's procedure. The fluorescence was determined at 488 nm excitation and 525/30 emission using a Guava EasyCyte HT System (Millipore, Bedford, MA, USA).

### 2.5. Terminal Deoxynucleotidyl Transferase dUTP Nick End-Labeling (TUNEL) Assay

Cells in the logarithmic phase were collected and cultured in 6-well plates at 3 × 10^5^ cells/well. After treatment, neuronal apoptosis was assessed by using an ApoBrdU DNA Fragmentation Assay Kit (BioVision, Mountain View, CA, USA), consistent with the manufacturer's instructions. The nuclei were counterstained with PI.

### 2.6. Confocal Microscopy

In a confocal dish, SK-N-SH cells were incubated in HBSS medium (Gibco, Grand Island, NY, USA) containing 5 *μ*M MitoSOX and washed three times with HBSS. The cells were imaged on a Zeiss LSM710 microscope equipped with a standard set of lasers through a 63x oil objective, installed at the Center for University Wide Research Facilities at Jeonbuk National University. The excitation wavelengths were 488, 543, and 633 nm. The bandpass filters were set at 500–550 (Alexa Fluor 488), 560–615 nm (Cy3, Alexa Fluor 568), and 650–750 nm (Alexa Fluor 647).

### 2.7. Cytosol and Mitochondrial ROS Assay

SK-N-SH cells were incubated in either HBSS containing 10 *μ*M 2′,7′-dichlorodihydrofluorescein diacetate (H_2_-DCFDA) at 37°C for 30 min or 5 *μ*M MitoSOX at 37°C for 10 min. Cells were transferred to a clear 96-well plate for flow cytometry analysis using a Guava EasyCyte HT System (Millipore, Bedford, MA, USA).

### 2.8. JC-1 Assay

SK-N-SH cells were incubated in HBSS containing 10 *μ*M JC-1 at 37°C for 30 min. Cells were transferred to a clear 96-well plate for flow cytometry analysis using a Guava EasyCyte HT System (Millipore, Bedford, MA, USA). JC-1-stained cells on coverslips were imaged on a fluorescence microscope (Nikon Eclipse 80i).

### 2.9. Calcineurin Activity Assay

The calcineurin cellular activity assay kit (Enzo Life Sciences #BML-AK816-0001, USA) was used consistent with the manufacturer's instructions to determine the phosphatase activity of calcineurin in SK-N-SH cells [39]. In brief, the cells were lysed on ice in lysis buffer containing protease inhibitors. Phosphatase activity was quantified by detecting free phosphate released from the reaction by measuring the absorbance of malachite green (OD 620 nm) using a SpectraMax M2 (Molecular Devices).

### 2.10. Western Blot Analysis

Cells in the logarithmic phase were collected and cultured in a 6-well plate at 3 × 10^5^ cells/well. The western blot method has been described in detail previously [20]. We used a nuclear/cytosol fractionation kit (#K266, BioVision). After treatments, cells were washed with PBS and lysed in lysis buffer (25 mM HEPES (4-(2-hydroxyethyl)-1-piperazineethanesulfonic acid), pH 7.4, 100 mM NaCl, 1 mM ethylene diamine tetra acetic acid (EDTA), 5 mM MgCl_2_, 0.1 mM dithiothreitol (DTT), and a protease inhibitor mixture). Equal quantities of proteins (more than 15 *μ*g/*μ*L) in the nuclear or cytosolic extracts were electrophoretically resolved on a 10% sodium dodecyl sulfate (SDS) poly-acrylamide gel and transferred to a nitrocellulose membrane. Immunoreactivity was detected through consecutive incubation with blocking solution using 5% skim milk and primary antibodies, followed by the corresponding horseradish peroxidase-conjugated secondary antibodies, and finally developed using enhanced chemiluminescence substances (i.e., west save gold detection kit (LF-QC0103, AbFrontier Inc.)). The primary antibodies (anti-calcineurin at a dilution of 1 : 1000 (ab109412, Abcam plc), anti-NFAT1 at a dilution of 1 : 1000 (#5861, Cell Signaling), anti-lamin A/C at a dilution of 1 : 10000 (ab108595, Abcam plc), and anti-*β*-actin at a dilution of 1 : 5000 (A5441, Sigma Aldrich)) were diluted with antibody solution (1% skim milk in TBST). Images were inspected using a Fusion FX7 imaging system (Vilber Lourmat, Torcy Z.I. Sud, France). Densitometry of the signal bands was evaluated using the Bio-1D software (Vilber Lourmat, Marne La Vallee, France).

### 2.11. Statistical Analysis

Results are expressed as the means ± standard error of the mean (SEM) from at least three independent replicates. All experiments were analyzed by the one-way analysis of variance (ANOVA). Comparisons of three or more groups were made using Tukey's posttests. All statistical analyses were implemented with GraphPad Prism version 5.0. *p* values (^∗^*p* < 0.05, ^∗∗^*p* < 0.01, or ^∗∗∗^*p* < 0.001) were considered statistically significant.

## 3. Results

### 3.1. Prion Peptide 106-126 Generated More Mitochondrial ROS than Cytosolic ROS

**Figure 1:**
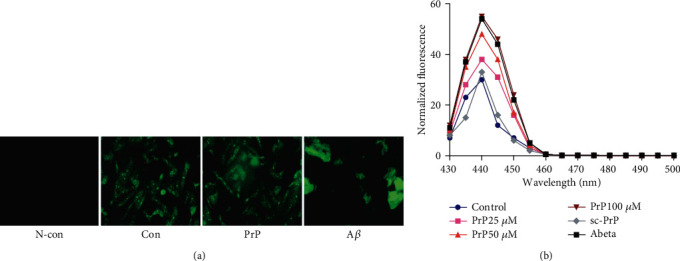
Prion peptide 106-126 aggregation status. (a) Neuroblastoma cells (SK-N-SH) were pretreated with 20 *μ*M ThT for 30 min and then exposed to 25, 50, and 100 *μ*M PrP 106-126, 100 *μ*M sc-PrP, or 100 *μ*M amyloid *β* for 6 h. (b) Fluorescence was evaluated by spectrum analysis and fluorescence microscopy.

Prion peptide 106-126 has previously been known to induce neurotoxicity as a result of its ability to form aggregates [46]. Thioflavin-T (ThT) binding was employed to confirm the amyloid fibrils formed by our PrP 106-126 peptides. Binding of ThT to polypeptide chains is specific for the cross-*β* structure of amyloid fibrils. We identified binding of ThT to prion peptide and amyloid *β* by fluorescence microscopy (Figure 1(a)). An increase in ThT fluorescence at 430–500 nm is observed, as shown in Figure 1(b), supporting a cross-*β* structure for PrP 106-126 fibrils.

**Figure 2:**
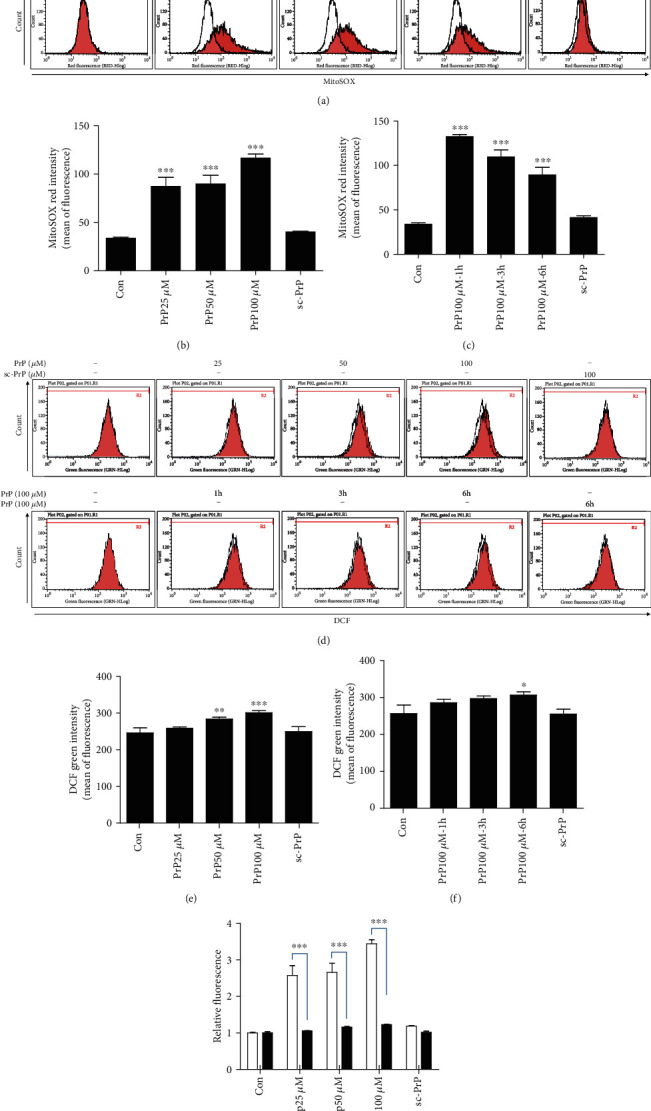
PrP (106-126) increased superoxide more than hydrogen peroxide. (a) Neuroblastoma cells (SK-N-SH) were exposed to PrP (106-126) or scrambled PrP in a dose- and time-dependent manner. Mitochondrial ROS was evaluated by a MitoSOX assay. (b, c) Bar graph showing the averages of the red fluorescence (MitoSOX) in a PrP dose- and time-dependent manner, respectively. Values represent the mean ± SEM (*n* = 10). ^∗∗∗^*p* < 0.001 vs. control. (d) SK-N-SH cells were treated with PrP (106-126) or scrambled PrP in a dose- and time-dependent manner. Cytosol ROS was evaluated by a DCF assay. (e, f) Bar graph showing the averages of the green fluorescence (DCF) in a PrP dose- and time-dependent manner, respectively. (g) Relative MitoSOX and DCF fluorescence in a PrP dose-dependent manner. Values represent the mean ± SEM (*n* = 10). ^∗^*p* < 0.05, ^∗∗^*p* < 0.01, and ^∗∗∗^*p* < 0.001 vs. control.

Although there is prior research that has suggested that prion peptide could induce cellular ROS in neurons, ROS's origin has not been examined. To determine this, we performed mitochondrial and cytosolic ROS detection experiments using both DCF and MitoSOX assays. PrP (106-126)-mediated mitochondrial ROS generation rose dose- and time-dependently in SK-N-SH neuroblastoma cells, whereas scrambled PrP did not increase mitochondrial ROS (Figures 2(a)–2(c)). PrP (106-126) slightly increased cytosolic ROS generation dose- and time-dependently (Figures 2(d)–2(f)). We determined that PrP (106-126) upregulated mitochondrial ROS more than cytosolic ROS (Figure 2(g)), suggesting that PrP (106-126) influences mitochondrial ROS and mitochondrial dysfunction in neuronal cells.

### 3.2. Prion Peptide 106-126 Promotes Neuronal Apoptosis through Mitochondrial ROS Generation

**Figure 3:**
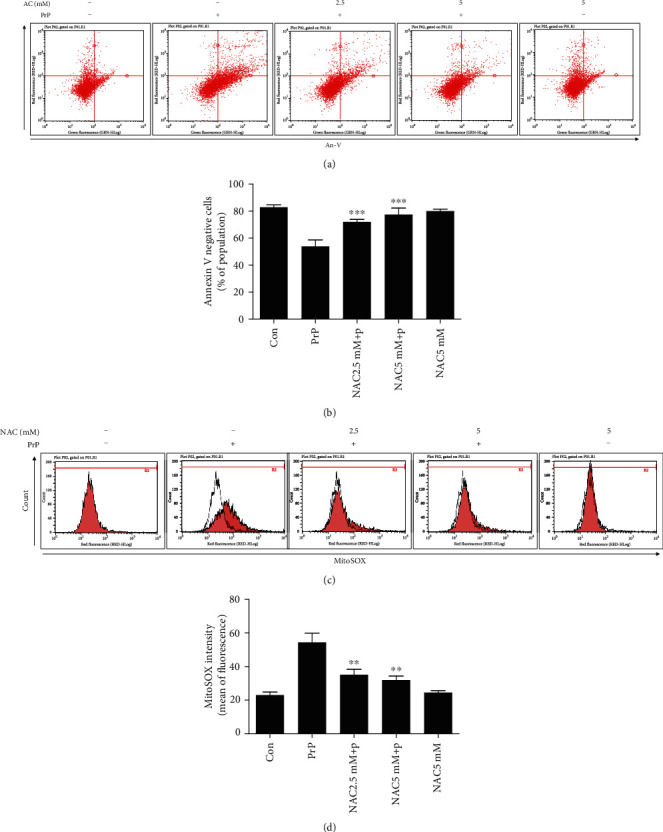
ROS scavenger treatment attenuated PrP (106-126)-mediated mitochondrial ROS and neurotoxicity. SK-N-SH cells were pretreated with NAC (*N*-acetyl-l-cysteine) (1 h) at 2.5 and 5 mM and then exposed to 100 *μ*M PrP (106-126) for 6 hours. (a) Cell viability was evaluated by an annexin V assay using FITC-annexin V, which combines with phosphatidylserine on the plasma membrane during the apoptotic processes. (b) Bar graph showing the averages of the annexin V-negative cells. Values represent the mean ± SEM (*n* = 10). ^∗∗∗^*p* < 0.001 vs. PrP. (c) Mitochondrial ROS was evaluated by a MitoSOX assay. (d) Bar graph showing the averages of the red fluorescence (MitoSOX). Values represent the mean ± SEM (*n* = 10). ^∗∗^*p* < 0.01 vs. PrP.

We examined whether PrP-induced mitochondrial ROS influenced neurotoxicity using the ROS scavenger NAC. NAC treatment attenuated PrP-mediated neuronal apoptosis dose-dependently (Figures 3(a) and 3(b)). We determined that NAC, as a reactive oxygen species (ROS) scavenger, decreased PrP-mediated mitochondrial ROS generation (Figures 3(c) and 3(d)), confirming that the prion peptide induces neuronal apoptosis through mitochondrial ROS generation.

**Figure 4:**
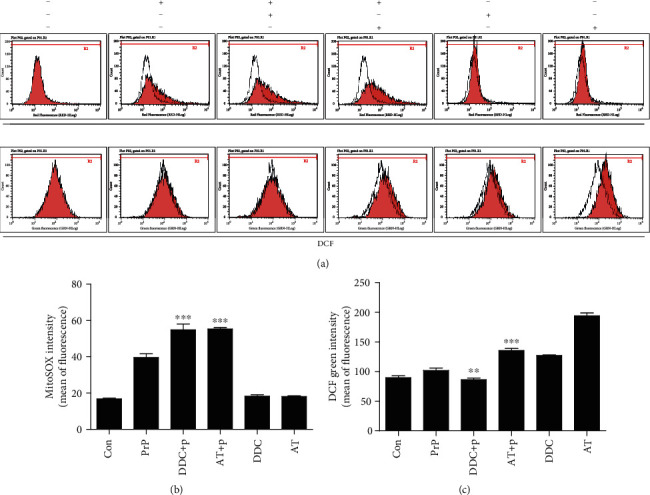
PrP (106-126) did not influence SOD or catalase enzyme. SK-N-SH cells were pretreated with DDC (diethyldithiocarbamate) (1 h) or AT (3-amino-1,2,4-triazole) (24 h) and then exposed to 100 *μ*M PrP (106-126) for 6 hours. (a) Mitochondrial ROS was evaluated by a MitoSOX assay, and cytosol ROS was evaluated by a DCF assay. (b) Bar graph showing the averages of the red fluorescence (MitoSOX). (c) Bar graph showing the averages of the green fluorescence (DCF). Values represent the mean ± SEM (*n* = 10). ^∗∗^*p* < 0.01, ^∗∗∗^*p* < 0.001 vs. PrP.

Sinclair et al. have suggested that the prion aggravates an apoptotic pathway through mitochondrial dysfunction and mislocalisation of SOD2 to cytosolic caspases [47]. We investigated the impact of PrP (106-126) on superoxide dismutases (SODs) and a catalase enzyme using an SOD inhibitor (diethyldithiocarbamate; DDC) and a catalase inhibitor (3-amino-1,2,4-triazole; AT). We determined that DDC and AT increased mitochondrial ROS, including superoxide, which had already been raised by PrP (106-126), meaning that this prion peptide did not influence SOD function (Figures 4(a) and 4(b)). DDC decreased cytosolic ROS such as hydrogen peroxide while AT increased cytosolic ROS that had already been raised by PrP (106-126) (Figures 4(a) and 4(c)), meaning that PrP (106-126) did not impair the SOD and the catalase. These results indicate that the prion peptide increased mitochondrial ROS production through pathways other than SOD and catalase impairment.

### 3.3. Prion Peptide Induced Neurotoxicity via CaN Activation

**Figure 5:**
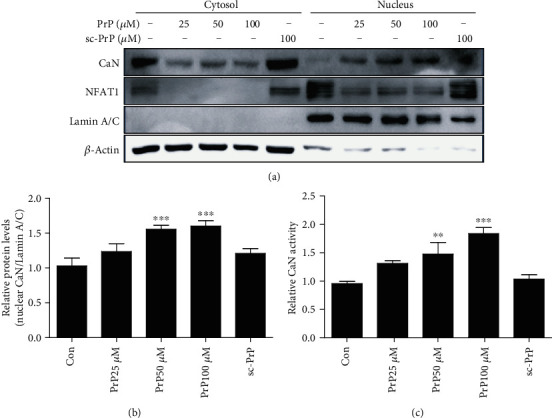
PrP (106-126) treatment upregulated calcineurin (CaN) activity. SK-N-SH cells were exposed to PrP (106-126) or scrambled PrP in a dose-dependent manner for 5 hours. (a) Cytosolic and nuclear fractions obtained from SK-N-SH cells induced for human calcineurin expression are analyzed by western blot with antibodies for CaN, for the cytosolic *β*-actin marker, and for the nuclear lamin A/C marker. (b) Bar graph representing the average nuclear CaN protein levels. The expression data were normalized to lamin A/C expression. The expression levels were evaluated by quantifying the protein bands, depicted by densitometric values beside the blot. Values represent the mean ± SEM (*n* = 5). ^∗∗^*p* < 0.01, ^∗∗∗^*p* < 0.001 vs. control.

In a previous study, we demonstrated how PrP (106-126) induces neurotoxicity through calcium alteration [40]. In this study, we checked whether PrP (106-126) alters CaN in neuronal cells. PrP (106-126) increased nuclear CaN translocation dose-dependently (Figures 5(a) and 5(b)). We identified CaN activation by prion peptide treatment using a CaN phosphatase activity assay (Figure 5(c)). We also identified NFAT1 as a transcriptional factor related to CaN, and PrP (106-126) decreased NFAT1 protein expression in both the nucleus and cytosol (Figure 5(a)). This result suggests that NFAT is not dependent on CaN.

**Figure 6:**
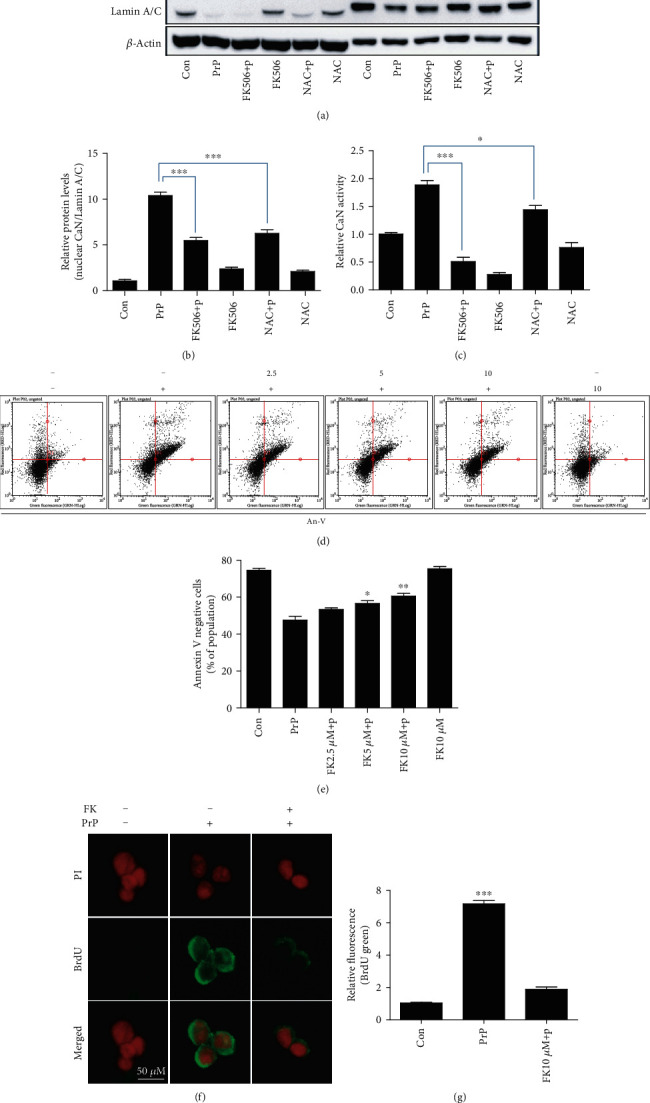
FK506 treatment attenuates PrP (106-126)-induced neurotoxicity via CaN inhibition. SK-N-SH cells were pretreated with FK506 (10 *μ*M, 1 h) or NAC (5 mM, 1 h) and then exposed to 100 *μ*M PrP (106-126) for 5 hours. (a) Cytosolic and nuclear fractions obtained from SK-N-SH cells induced for human calcineurin expression are analyzed by western blot with antibodies for CaN, for the cytosolic *β*-actin marker, and for the nuclear lamin A/C marker. (b) Bar graph representing the average nuclear CaN protein levels. The expression data were normalized to lamin A/C expression. (c) CaN activity was evaluated by a CaN activity assay. Values represent the mean ± SEM (*n* = 5). ^∗^*p* < 0.05, ^∗∗∗^*p* < 0.001 vs. PrP. (d) SK-N-SH cells were pretreated with FK506 (1 h) in a dose-dependent manner and then exposed to 100 *μ*M PrP (106-126) for 6 hours. Cell viability was evaluated by an annexin V assay using FITC-annexin V, which combines with phosphatidylserine on the plasma membrane during apoptotic processes. (e) Bar graph showing the averages of the annexin V-negative cells. Values represent the mean ± SEM (*n* = 10). ^∗^*p* < 0.05, ^∗∗^*p* < 0.01 vs. PrP. (f) TUNEL-positive (green) immunofluorescence images were obtained after exposure to 100 *μ*M PrP (106-126) (6 h) in the absence or presence of FK506 (10 *μ*M, 1 h). The cell nuclei were counterstained with PI (red). (g) Bar graph showing the relative mean values of the green fluorescence (BrdU). Values represent the mean ± SEM (*n* = 5). ^∗∗∗^*p* < 0.001 vs. control.

PrP (106-126)-increased CaN was reduced by the CaN inhibitor FK506 and ROS scavenger NAC (Figures 6(a) and 6(b)). CaN activity was also decreased by FK506 and NAC (Figure 6(c)). NFAT1 is decreased in PrP-treated cells, and FK506 also decreased NFAT1 protein expression (Figure 6(a)). Based on these results, we suggest that CaN and ROS influence each other through a feedback loop. To investigate the effect of prion peptide-mediated CaN activation on neuronal apoptosis, we employed the CaN inhibitor FK506. We found that FK506 attenuated prion peptide-induced neuronal apoptosis dose-dependently using an An-V/PI assay (Figures 6(d) and 6(e)). In addition, we identified that FK506 repressed PrP-mediated DNA strand damage using a TUNEL assay (Figures 6(f) and 6(g)). We suggest that PrP (106-126) induces neuronal apoptosis through CaN activation.

### 3.4. CaN Activation by Prion Peptide Promoted Mitochondrial Dysfunction

**Figure 7:**
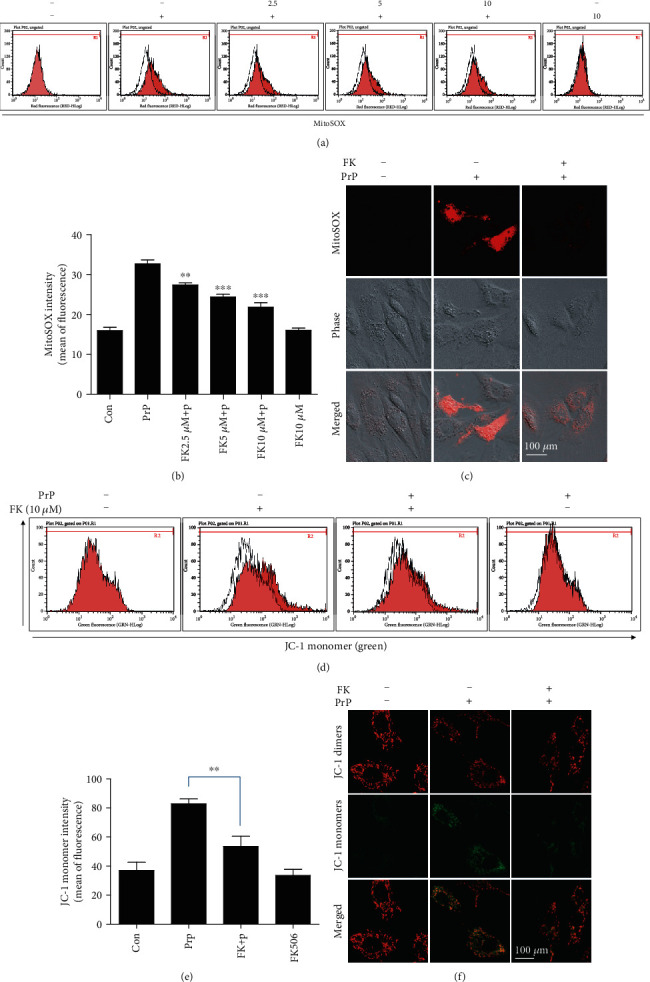
PrP (106-126)-mediated calcineurin activation induced neurotoxicity via mitochondrial dysfunction. SK-N-SH cells were pretreated with FK506 (1 h) and then exposed to 100 *μ*M PrP (106-126) for 6 hours. (a) Mitochondrial ROS was evaluated by a MitoSOX assay. (b) Bar graph showing the averages of the red fluorescence (MitoSOX). Values represent the mean ± SEM (*n* = 10). ^∗∗^*p* < 0.01, ^∗∗∗^*p* < 0.001 vs. PrP. (c) MitoSOX fluorescence images were obtained after exposure to 100 *μ*M PrP (106-126) (6 h) in the absence or presence of FK506 (10 *μ*M, 1 h). (d) Mitochondrial membrane potential was evaluated by a JC-1 assay using flow cytometry. In green fluorescent colors, JC-1 accumulates as green monomers in the mitochondria of cells with impaired mitochondrial membrane potential function. (e) Bar graph showing the averages of the green fluorescence (JC-1 monomers). Values represent the mean ± SEM (*n* = 10). ^∗∗^*p* < 0.01 vs. PrP. (f) JC-1 fluorescence images were obtained after exposure to 100 *μ*M PrP (106-126) (6 h) in the absence or presence of FK506 (10 *μ*M, 1 h).

To investigate the effect of prion peptide inducing CaN activation on mitochondrial function, we ran an experiment using the MitoSOX and JC-1 assay using FK506. We used the MitoSOX assay to determine that treatment with FK506 decreased the mitochondrial ROS in PrP-treated neuronal cells (Figures 7(a) and 7(b)). We also imaged the MitoSOX using confocal microscopy (Figure 7(c)). Further, we determined that FK506 repressed the prion peptide-induced mitochondrial dysfunction using the JC-1 assay (Figures 7(d) and 7(e)) and fluorescence microscopy (Figure 7(f)). These results demonstrate that prion peptide-mediated neuronal apoptosis is dependent on CaN activation and mitochondrial ROS and that CaN is a key regulator of prion peptide-mediated ROS generation and neurotoxicity. In sum, our results suggest that prion peptide (106-126) induces mitochondrial ROS and neuronal apoptosis through CaN activation in neuronal cells.

## 4. Discussion

Neurotoxic PrP 106-126 preserves biochemical substances similar to those of the abnormally folded prion protein PrPsc, together with protease resistance, *β*-sheet construction, and cytotoxicity [13, 48–50]. Previous literature has investigated prion pathogenesis and neurotoxic pathways using Rocky Mountain Laboratory (RML) strain and antibody-derived anti-PrP ligands in *in vivo* models [51–53]. Typical neurodegenerative fluctuations in prion diseases have been observed in the absence of detectable PrPsc, suggesting that prion disorders are caused by alternative mechanisms of neuronal damage, as well as PrPsc [54, 55]. PrP 106-126 has been suggested as one such alternative contributor to the pathogenic and molecular properties of PrPsc [56]. Although the functional and mechanical properties of prion protein remain unclear, a number of studies have suggested that prions are triggered by accumulation in the brain of amyloid plaque PrPsc, similar to amyloid-*β* in Alzheimer's disease [5, 57, 58].

CaN was recently suggested as an important therapeutic target for the potential treatment of neurodegenerative diseases [59, 60]. CaN is abundant in the synaptic terminals and cytosol of neurons, which suggest that it may play a critical role in the maintenance of cellular homeostasis during calcium alteration [44, 45]. Hyperactivation of CaN is implicated in a reversible neuronal apoptotic pathway involving Bcl-2 family proteins [61, 62]. Moreover, CaN plays an essential role in modulating gene expression, including cAMP-response element binding protein (CREB) and nuclear factor of activated T-cell (NFAT), as well as regulating calcium alteration [44, 63–65]. Since phosphorylated CREB and dephosphorylated NFAT4 translocate into the nucleus and induce gene expression, several studies have observed CaN protein expression in nuclear fraction [66–68]. Consistent with these studies, we identified translocation of CaN into a nucleus caused by prion peptide treatment in neuronal cells (Figure 4). Other literature has suggested that CaN is activated by cleavage of the autoinhibitory domain that transforms it into a constitutively active form [69–71]. We could not observe any meaningful cleavage form CaN in prion peptide-treated neuronal cells (not shown). Accordingly, we suggest that prion peptide may induce CaN activation by nuclear translocation. However, the association between CaN and neurodegenerative progression remains contentious [72–74].

In our previous study, we prove that prion peptide 106-126 induced neurotoxicity through the AMPK-CaN-autophagy pathway [39, 40]. AMPK has been recognized in the past few years to act as a crucial integrator of autophagy and mitochondrial homeostasis by controlling various aspects of the cellular life cycle [75]. In this study, our goal is to investigate what kind of ROS is generated in prion-mediated neurotoxic conditions and elucidate the relationship between this ROS and CaN pathway in prion diseases. In future, further studies are required to investigate the role of AMPK in this mitochondrial ROS generation as well as apoptosis in prion diseases.

We observed that the expression of NFAT1 was decreased in the nucleus in PrP-treated condition (Figure 5(a)). CaN is well known to the phosphatase that promotes NFAT nuclear import, and its activity is regulated not only by upstream Ca^2+^ and calmodulin but also by multiple endogenous calcineurin inhibitors [76]. However, the specific role of each NFAT member in gene transcription during the cell cycle and apoptosis is not fully clear, especially in neurons [77]. In our results, NFAT1 alteration was not dependent on the CaN (Figures 5 and 6), but rather the total amount of NFAT1 of whole cell lysates was decreased in PrP-treated cells. Further studies will be needed to discover how prion peptide decreased NFAT1 protein expression in cellular pathways.

We demonstrated ROS generation using the MitoSOX and DCF assay. A MitoSOX usually indicates mitochondrial ROS, and DCF indicates cytosolic ROS. In Figure 3(c), AT alone induces DCF generation as compared to control. AT was used as a catalase inhibitor that prevents hydrogen peroxide to water (H_2_O) substitution. DDC and AT affected PrP-induced DCF generation. However, we focused on PrP-induced mitochondrial ROS (MitoSOX) because DCF generation by prion peptide was insignificant.

Several studies have reported that CaN activity is regulated by Ca^2+^ as well as by oxidative stress conditions [45, 78]. Accordingly, we investigated the relationship between CaN activity and intracellular ROS. Prior literature has suggested that PrP 106-126 induces intracellular ROS [31, 79].

## 5. Conclusion

Our results prove that PrP 106-126 generates more mitochondrial ROS than cytosolic ROS and PrP-mediated CaN activation regulated mitochondrial ROS in neuronal cells. These results also suggest that PrP-induced mitochondrial ROS production triggers CaN activation partially as circulating feedback action, and the regulation of CaN may be a practical therapeutic treatment for prion disease.

## Data Availability

The data used to support the findings of this study are available from the corresponding author upon request.
